# Parental Stress, Depression, Anxiety, and Participation in Neonatal Care in a Referral Brazilian NICU over Different Phases of the COVID-19 Pandemic

**DOI:** 10.3390/children12040496

**Published:** 2025-04-12

**Authors:** Dafne Barcala Gomez, Emanuelle Pessa Valente, Paolo Dalena, Ilaria Mariani, Suely Arruda Vidal, Maria Júlia Gonçalves Mello, Geyse Lima, Juliana Barradas Souza, Waldemar Brandão Neto, Vanessa Tenório Rodrigues, Bruna Malta Castro, Maria Luísa Pessoa, Eduarda Medeiros Cisneiros, Marzia Lazzerini

**Affiliations:** 1 Instituto de Medicina Integral (IMIP), Recife 50070-550, PE, Brazil; 2 Ospedale San Polo, Azienda Sanitaria Universitaria Giuliano Isontina (ASUGI), 34074 Monfalcone, Italy; 3 Institute for Maternal and Child Health IRCCS “Burlo Garofolo”, 34137 Trieste, Italy; 4 University of Trieste, 34127 Trieste, Italy; 5 Nursing Faculty Nossa Senhora das Graças, Universidade de Pernambuco, Recife 50100-010, PE, Brazil; 6 Maternal Adolescent Reproductive and Child Health Care Centre, Faculty of Epidemiology and Population Health, London School of Hygiene & Tropical Medicine, London WC1E 7HT, UK

**Keywords:** stress, depression, anxiety, parental participation, neonatal intensive care unit, COVID-19

## Abstract

**Background/Objectives:** During the COVID-19 pandemic, neonatal care units had to change protocols, and little is known about its impact on parents’ mental health. This study aimed to evaluate parental stress, depression, anxiety, and participation in neonatal care in a Brazilian neonatal intensive care unit (NICU) and observe changes over two different periods of the COVID-19 pandemic. **Methods:** Cross-sectional study comparing stress, depression, anxiety, and participation in neonatal care levels between two time periods: T1, high COVID-19 incidence (May 2020 to July 2020 and March 2021 to June 2021) and T2, low COVID-19 incidence (August 2020 to February 2021 and July 2021 to December 2021). High COVID-19 incidence was considered more than 40 confirmed COVID-19-related deaths/day. Validate tools used were the Parental Stressor Scale in NICU (PSS:NICU); the Edinburgh Postnatal Depression Scale (EPDS); the Edinburgh Postnatal Depression Scale-Anxiety subscale (EPDS-A); the State-Trait Anxiety Inventory (STAI); and the Index of Parental Participation (IPP). Stress level was pre-defined as the primary outcome. **Results**: 106 parents (98 mothers, 8 fathers) and 111 newborns were included. Overall, 51.9% of parents had a PSS:NICU score ≥ 3 (relevant stress level), 28.3% had an EPDS-A ≥ 6 (indicating anxiety), and 33.0% had an EPDS > 13 (indicating depression). At least one condition was present in 69 (65%) parents, while the three conditions were simultaneously observed in 17 (16%) parents. No significant differences were observed in the frequencies of stress, depression, or anxiety between the two periods. However, median stress occurrence level (SOL) was higher in T1 when compared to T2 (3.24 vs. 2.68; *p* = 0.02), mainly due to “Parental role alteration” (3.80 vs. 3.17; *p* = 0.046). The level of parental participation was not different between the two time periods (*p* = 0.23). Correlations between stress and both depression and anxiety scores were weak. Parental participation was not significantly correlated to other scores. **Conclusions:** Elevated levels of stress, depression, and anxiety were observed among NICU parents during both high and low COVID-19 incidence periods. High COVID-19 incidence seems to have particularly influenced stress levels related to parental role alteration. These findings highlight the importance of regularly assessing parental mental health in NICU settings.

## 1. Introduction

Neonatal intensive care unit (NICU) hospitalization is described as an overwhelming experience for parents, often associated with negative feelings and emotional distress for family members [1]. Worldwide, studies have reported high incidence of adverse mental health conditions—such as depression, anxiety, and stress—among parents experiencing NICU hospitalization [2]. These findings are particularly concerning given the critical role parents play in their infant’s care during NICU stay [3]. Moreover, poor parental mental health may negatively impact parent-infant bonding and have lasting implications for the child’s neurodevelopmental outcomes, both in the short and long term [4].

As neonatal care has advanced in recent decades, greater emphasis has been placed on supporting family presence in the NICU and empowering parents to participate in their infant’s care and decision-making processes [5,6]. Active parental involvement not only enhances satisfaction and confidence in caregiving roles but also contributes to improving their mental health outcomes [5]. Given these demonstrated benefits, family-centered care (FCC) has been increasingly recognized as a fundamental component of high-quality neonatal intensive care [6].

Even though unrestricted parental access to the NICU has been well established as an important factor for infant recovery and development, the COVID-19 pandemic imposed significant changes in visiting protocols [7,8]. To prevent SARS-CoV-2 transmission, NICUs adopted restrictive measures such as limiting physical contact with newborns, requiring visitors to use personal protective equipment (PPE), and even enforcing complete isolation [8]. These restrictions exacerbated challenges experienced by NICU parents, compounding existing stressors with pandemic-related burdens [9,10].

Studies indicate that maternal mental health was particularly affected during COVID-19 pandemic [9] raising concerns on how parents coped with NICU hospitalization under these unprecedented circumstances [10]. Research across diverse settings consistently demonstrated mental health impairment among NICU parents during the pandemic period [10,11]. For instance, a US study conducted across NICUs of 38 different states, reported parents suffered with emotional struggles, isolation feelings, lack of family-centered care, and deep disappointment with system-level decisions during their infant’s recovery [12]. Similarly, a Colombian qualitative study, highlighted parents’ struggles with limited newborn interaction, COVID-19 related fears and reduced social support [13]. Similar findings were described in Brazil, where a study in a public maternity hospital in Paraíba found that mothers of preterm infants faced social isolation, with profound social, emotional, and psychological consequences [14].

In Brazil, the COVID-19 cases started spreading by March 2020, followed by several control and prevention measures taken by the local health authorities [15]. For instance, the Brazilian Ministry of Health recommended suspending NICU visits by extended family (grandparents, siblings, and other people in the support network), only allowing access to asymptomatic mothers or fathers [16]. The pandemic also negatively influenced the daily lives of Brazilian families who accompany NICU hospitalization, impacting family dynamics, parental self-care, and infant care practices [11]. Furthermore, the impact on Brazil’s healthcare system exacerbated pre-existing regional disparities, particularly regarding shortages of healthcare professionals and NICU beds, affecting mostly the North and Northeast regions [11,14].

Assessing different settings is important, as some local aspects can influence outcomes, including parental mental health [17]. A recent review of the literature showed that several common risk factors in Brazil, such as low socioeconomic status, exposure to violence, and previous history of psychiatric disorders, were associated with depression and anxiety during pregnancy and the postpartum period [18]. The same study indicated that the prevalence of postnatal depression ranged from 12.5% to 38.8% [18]. Brazilian data on parental mental health is poor and shows a wide variation, which may reflect existing regional differences [18]. In 2011, a study with mothers of preterm infants in the North region of Brazil showed that 81.7% had a state of anxiety, 70% had trait anxiety, and 56.4% reported high levels of depressive symptoms [19], demonstrating the urgent need to address parental mental health in Brazilian NICUs.

This study is part of a multi-country cross-sectional study called “Empowering Parents in the NICU” (EPINICU) [17]. Previous EPINICU findings from an Italian NICU showed no significant impact of the COVID-19 pandemic on parental stress, depression, or participation in care in their setting [20]. However, in Brazil, few qualitative data were reported on NICU parents experiences during the COVID-19 pandemic, suggesting an existing knowledge gap [11,14,21]. Although NICU stressors have been described long before the COVID-19 pandemic and certainly will persist beyond it, acknowledging the COVID-19 pandemic burden on parental mental health is an important step toward preventing future parent–child separation events. More evidence on this topic may be of interest to researchers, policymakers and clinicians and may support further improvement in the quality of newborn care. The present study aimed at exploring levels of stress, anxiety, depression, and participation to care among NICU parents in a referral hospital at Pernambuco, the Brazilian northeast region, during different periods of the COVID-19 pandemic and to assess the correlation between those levels.

## 2. Materials and Methods

### 2.1. Study Design and Setting

This was a cross-sectional study, reported according to the Strengthening the Reporting of Observational Studies (STROBE) in Epidemiology guidelines [22] (Supplementary Table S1). The study compared the high COVID-19 incidence period (T1), covering the periods from May 2020 to July 2020 and March 2021 to June 2021, and the low COVID-19 incidence period (T2), covering the periods of August 2020 to February 2021 and July 2021 to December 2021. It was conducted at a Level III NICU (i.e., it provides intensive care to severely ill infants, including surgical and sub-specialty services) of the Institute of Integral Medicine Professor Fernando Figueira (IMIP), a university-affiliated hospital located in the Northeast of Brazil. The maternity was chosen as the referral facility for pregnant women and newborns affected by COVID-19 in Pernambuco. Due to restrictive measures, NICU admissions were reduced from an average of 110 to 80 NICU admissions/month, as well as the number of NICU beds (from 50 to 30 beds) and the Kangaroo Mother Care (KMC) nursery (from 20 to 10 beds).

To assess COVID-19 periods in Pernambuco, we used data from the Institute for Risk and Disaster Reduction of Pernambuco [23] and from the Brazilian Ministry of Health [24]. The high COVID-19 incidence period (T1) was considered >40 confirmed COVID-19-related deaths/day, coexisting with government restrictive measures, and included periods from May 2020 to July 2020 and March 2021 to June 202. The low COVID-19 incidence period (T2) covered the remaining months, August 2020 to February 2021 and July 2021 to December 2021.

### 2.2. Study Participants

Parents of newborns hospitalized at NICU or semi-intensive units for at least 24 h who were at least 18 years old and fluent in Portuguese were prospectively enrolled. Exclusion criteria were death of the newborn or mother at any period of the study and the presence of severe mental disability or illiteracy that could potentially affect self-completing the questionnaires.

### 2.3. Study Outcomes and Data Collection Tools

Parental stress, depression, and anxiety were all assessed with Brazilian versions of the validated tools (Supplementary Table S2). Stress was assessed with the Parental Stressor Scale in NICU (PSS:NICU) [25], which contains 26 items distributed in 3 domains: (1) sights and sounds; (2) infant behavior and appearance; and (3) parental role alteration. Stress is rated with a Likert scale from 1 point (“not at all stressful”) to 5 points (“extreme/severe stress”) with a “not applicable” (N/A) option. Total scores can be calculated including only experienced items (Stress Occurrence Level [SOL]) or scoring “not applicable items” with one point (Overall Stress Level [OSL]) [26]. In our study, SOL was chosen to evaluate the frequency of stress because it is the most reliable way to evaluate the stress directly related to the NICU experience, since it excludes the “not experienced” items [25], while OSL focus is the NICU environment [26]. In the absence of a clear indication in the existing literature, we defined “high stress” as a median SOL score ≥ 3 [17,20].

Parental depression was assessed by the Edinburgh Postnatal Depression Scale (EPDS), validated in Portuguese [27]. Respondents rated symptoms in the past seven days using a Likert scale from “0” = “not at all” to “3” = “most of the time/quite often”, resulting in a final score from 0 to 30 [27]. Using the cutoff point of 13, the scale has a high positive predictive value for diagnosing postpartum depression [27].

To assess anxiety, the State-Trait Anxiety Inventory (STAI) includes two different scales: STAI-Y1 evaluates the current state of anxiety, and the Trait Anxiety Scale (STAI-Y2) evaluates anxiety proneness. Therefore, STAI-Y2 evaluates individual differences, relatively stable personality traits, in the tendency to react with an increase in the state of anxiety in the face of situations perceived as threatening. The 20-item scale is evaluated with a Likert scale ranging from 1 point “almost never” to 4 points “almost always”. Scores over 40 were indicative of “any anxiety”, scores in between 41 and 50 “mild anxiety”, 51–60 “moderate anxiety”, and >60 “severe anxiety”. Although the EPDS was initially developed for screening postpartum depression in community settings [28], several studies have assessed the potential utility of a subset of items to identify women with elevated anxiety symptoms [29]. In this study, we chose to measure anxiety trait with STAI-Y2 (STAI-trait) and anxiety, in general, with the EPDS-Anxiety subscale (EPDS-A) [29].

Parental participation in neonatal care was measured with the Index of Parental Participation (IPP) [30], slightly adapted by authors for use in NICU, in agreement with the original author and submitted to cross-cultural adaptation, translation, and content validation (Supplementary Figure S1). The IPP includes 4 subdomains: activities related to daily living; providing comfort; advocating for newborn health; and technical tasks. Each item was scored dichotomously (yes [1] or no [0]) according to the parent’s experience in the last 24 h. The score ranges from 0 to 30, with a higher total score representing higher participation; however, no cutoff has ever been established.

Demographic and clinical information was collected with a structured form developed by authors with predefined case definitions and field-tested before use. For mothers or fathers: age, marital status, place of residence, educational status, occupation, family income (minimum wage, established by Brazilian government during study period) [31], and number of people that offer support at home. For newborns, it included gestational age at birth (as described on the medical record), Apgar at 5′, resuscitation at birth (considered use of positive pressure ventilation), presence of severe malformation (with clinical impairment), admission unit (intensive care or semi-intensive care), orotracheal intubation at any time, parenteral nutrition, surgery procedure, COVID-19 status (suspected, patient collected nasopharyngeal swab for COVID-19 or confirmed, positive result for COVID-19 swab), and length of hospitalization in days.

### 2.4. Data Collection Procedures

Data was collected between May 2020 and December 2021. Self-administered, anonymous questionnaires in Portuguese were presented to parents at least 24 h after admission by trained research collaborators. Clinical data were collected from medical records, and demographic data from parents were completed by interview. Data was inserted into REDCap, into a digital database developed ad hoc, following standard operational procedures.

### 2.5. Data Analysis

#### Sample Calculation

Considering stress level as our primary outcome, assuming as clinically relevant a difference in the mean of the PSS:NICU stress occurrence level score (SOL) of 0.5 points between time periods, a 2:1 sample of 31 parents for group one and 63 parents for group two was calculated. A standard deviation of 0.8 in both groups, with an alpha of 0.05 and a power of the test equal to 80%, was considered.

Categorical variables were described in absolute and relative frequency distribution tables, and for quantitative variables, the central trend measures expressed in means and standard deviation (SD) or median and interquartile interval (IQR) were calculated. To calculate the PSS:NICU scores in each question, we used the median of the Likert scale according to SOL and OSL.

The following cut-offs were used as suggested by literature: a relevant level of stress was considered if median PSS: NICU was ≥3 [25]; depression if EPDS ≥ 13 [27]; anxiety if STAI-Y2 state > 40 [32] or EPDS-A ≥ 6. Odds ratios (OR) and adjusted odds ratios (aOR) were calculated, with 95% confidence intervals (CI) and *p*-values of significance. Correlation between scores was analyzed using Spearman’s rank correlation coefficients, assuming +1 indicating a positive correlation and −1 indicating a negative correlation and zero indicating no association. Correlation strengths were interpreted as follows: ρ < 0.3 = weak; 0.3 ≤ ρ < 0.7 = moderate; ρ ≥ 0.7 = strong.

The difference between periods T1 and T2 was assessed considering a two-sample *t*-test and the Wilcoxon-Mann–Whitney test. All the tests were two-tailed, and *p*-values less than 0.05 were considered statistically significant. Statistical analyses were performed using R version 4.3.1.

### 2.6. Ethical Considerations

The research was conducted in accordance with the Declaration of Helsinki and approved by the Ethical Committee in Brazil (Comissão Nacional de Ética em Pesquisa [CONEP]) number 3931201, May 2020. Before data collection, participants were informed of the objectives and methods of the study, including their rights in declining participation. An informed written consent was signed by each participant. Anonymity in data collection was ensured by not collecting information that could disclose participants’ identity. Data transmission and storage were secured by encryption.

## 3. Results

During this study, 676 newborns were admitted to the NICU, and 443 (65.5%) met inclusion criteria. Overall, 111 newborns and 106 parents (98 mothers, 8 fathers) were included, 68 in T1 (high COVID-19 incidence) and 38 in T2 (low COVID-19 incidence) (Supplementary Figure S2).

### 3.1. Patients’ Characteristics

Demographic parent characteristics and newborn clinical characteristics are reported in Table 1 and Table 2.

Most of the mothers, 75.5%, reported a stable union or being married, and 61.3% were unemployed or “housewives”. No statistical differences between the periods were found regarding parental demographic characteristics, except for years of education (>8 years of education, T1 69.0% vs. T2 94.3%; *p* = 0.07) (Table 1).

Regarding newborns (Table 2), 73.9% were preterm infants, 28.8% had severe malformation, and 91.9% required intensive care, characterizing a population of high-risk neonates. A low incidence of positive COVID-19 was found among the neonates, as reported in other centers during the pandemic [33]. The mean length of hospital stay was 25 days, similar in both periods.

### 3.2. Stress, Anxiety, Depression, and Parental Participation

**Figure 1:**
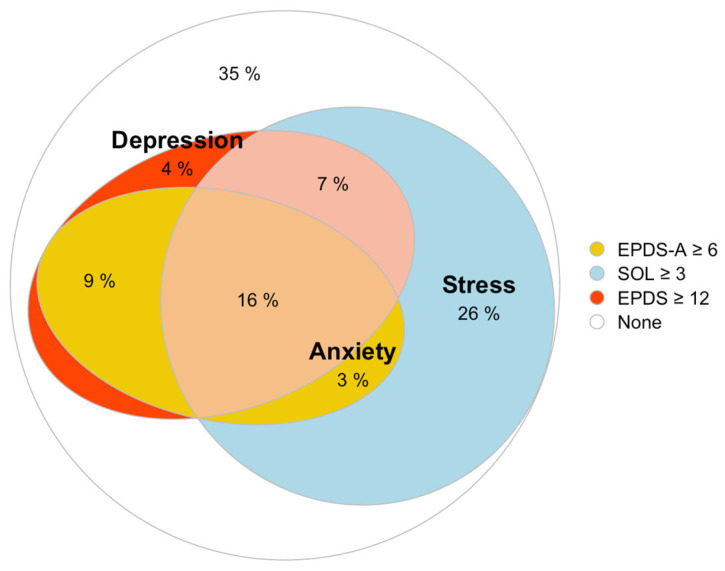
Frequency of parental depression (EPDS ≥ 12), anxiety (EPDS-A ≥ 6), and stress (SOL ≥ 3) and intersections among identified populations.

At least one condition was present in 69 (65%) parents, while the three conditions were simultaneously observed in 17 (16%) parents (Figure 1).

Regarding the PSS:NICU results presented in Table 3, 51.9% of parents had stress occurrence, SOL ≥ 3 and no difference was found between the two periods (T1 56.3% × T2 42.9%, *p* = 0.298). However, “Parental role alteration” (*p* = 0.046) and SOL median scores (*p* = 0.021) were both found significantly higher in T1 period.

Depression and anxiety frequencies did not show any significant difference during T1 and T2. Trait anxiety was identified in 66.3% (*n* = 69) of parents, and the EPDS-A score detected anxiety in 28.3% (*n* = 30) of parents. Depression (EPDS > 13) was present in 33.0% (*n* = 35) of the full sample (T1 = 33.8%, *n* = 24 vs. T2 = 31.4%, *n* = 11; *p* = 0.999).

The IPP median score was 19.39 [15.0, 24.7] and did not differ in the two periods (T1 = 20 [13.5, 24] vs. T2 = 23 [18.0, 25.0]; *p* = 0.235); also, no significant difference was observed in any of the four categories of participation.

**Figure 2:**
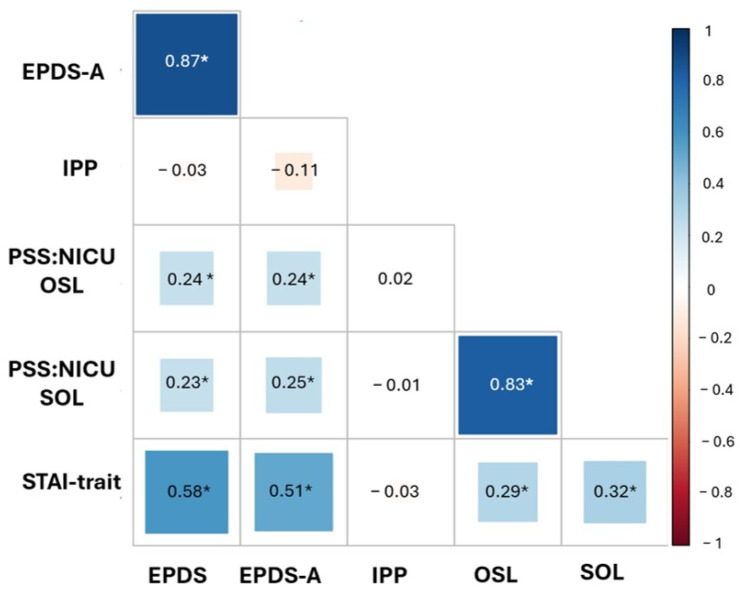
Spearman’s rank correlation coefficients between scores from PSS:NICU, Parental Stressor Scale in the NICU; SOL, Stress Overall Level; OSL, Overall Stress Level; STAI= State-Trait Anxiety Inventory; EPDS, the Edinburgh Postnatal Depression Scale; EPDS-A = Edinburgh Postnatal Depression Scale Anxiety; IPP, Index of Parenteral Participation. * Significant correlations (*p* < 0.05).

While statistically significant, the correlations between stress (SOL) and both depression (ρ = 0.23, *p* = 0.016) and anxiety-trait (ρ = 0.32, *p* < 0.001) were weak. In contrast, a strong positive correlation was observed between STAI-trait and both EPDS-A scores (ρ = 0.51, *p* < 0.001) and EPDS (ρ = 0.58, *p* < 0.001). Parental participation (IPP) showed no significant correlations with any of the measured psychological outcomes (PSS:NICU-OSL, PSS:NICU-SOL, EPDS, EPDS-A, or STAI-trait), as illustrated in Figure 2.

## 4. Discussion

This is the first Brazilian study to report NICU parents mental health and participation in neonatal care during the first two years of the COVID-19 pandemic. Stress, anxiety, depression, and parental participation during their newborn hospitalization were measured using validated tools. The study findings highlight that, comparing periods of high (T1) and low (T2) incidence of COVID-19, median scores of stress occurrence levels were higher during T1. However, no significant difference was observed in the frequency of parental stress, depression, and anxiety. Surprisingly, parental participation scores did not differ, and results showed that some level of participation was reported during the pandemic period.

The NICU experience is already known to have an impact on parental mental health [34]. However, the COVID-19 pandemic raised concern on increasing psychological distress in this already vulnerable group [9]. Attempting to mitigate the spread of SARS-CoV-2, visitation protocols changed, impacting how family-centered care was practiced. Less parental participation in neonatal care, less communication with NICU staff, and suspension of support programs were reported [7,9].

Even though parents presented alarming levels of stress, anxiety, and depression in our study, existing literature prior to the pandemic period does not distance itself from our results [35]. Among 39 included studies in a recent meta-analysis, mothers presented rates of stress ranging from 23% to 76%, anxiety from 13% to 93%, and depression from 18% to 52%, demonstrating a wide variation among different populations [35].

Our study found that more than half of participating parents reported moderate to high stress levels. While cross-study comparisons remain challenging due to heterogeneity in PSS:NICU scoring thresholds and interpretation criteria [36], our results are consistent with international findings. Notably, an Egyptian multicenter study (*n* = 743) reported even higher stress prevalence, with 75% of parents experiencing high stress levels [37]. Identified stress correlates in that population included mechanical ventilation, previous neonatal death, parents living far from hospitals, cesarean delivery, low parental income, and hospitalization exceeding five days [36]. Although we did not formally assess the correlation between neonatal and parental characteristics with stress, our cohort’s composition—predominantly featuring high-risk neonates—suggests this population may represent a particularly vulnerable subgroup for parental stress [37].

Our study population demonstrated an overall SOL median score of 3.05, which was slightly elevated compared to the median score of 2.86 reported by Kegler et al. in their study of 127 mothers and 77 fathers in a Brazilian NICU [38]. “Parental role alteration” emerged as the main source of stress among our participants, aligning with Kegler’s findings [37]. Also, a recent meta-analysis that included 53 studies from five continents underscored the impact of parental role alteration as a major contributor to parental stress worldwide. However, it is notable that only one study from South America was included [35]. In our study we identified a significantly higher score for “Parental role alteration” during T1, reinforcing the effects of the COVID-19 pandemic with restrictive policies, such as mother-newborn separation, on parental mental health [36].

During periods of higher incidence of COVID-19 cases, restrictive measures were intensified [24]. In our NICU, fathers were prohibited from entering, mothers were only allowed to enter the unit if they did not have any symptoms, and wearing a mask was mandatory. The mother-baby binomial was often separated due to maternal illness or even socioeconomic difficulties such as not being able to afford transportation to the hospital. Brazilian data reported increased financial insecurity during the pandemic, with increased unemployment rates, resulting in deepening socioeconomic, racial, and ethnic inequalities [14]. All these factors may explain why stress levels were higher in T1 when compared to T2.

Despite evidence from Brazilian studies indicating an increase in postpartum depression and anxiety symptoms during the COVID-19 pandemic [39], these levels remained stable throughout both periods of our study. Research also indicates that anxiety and depression are closely related to socioeconomic factors [18]. Our study was conducted in a public health system setting, and as expected, we observed that more than half of the parents earned less than the minimum wage and were unemployed or housewives. Comparing our results with previous Brazilian literature is challenging due to the wide variability in findings and the fact that most prior Brazilian studies were conducted in regions with higher income concentration [18].

Furthermore, the provision of social and psychological support to mothers and the availability of Kangaroo Mother Care (KMC) can also influence results [18,40]. Among the various FCC strategies, Kangaroo Mother Care (KMC) is a particularly effective, low-cost intervention with significant potential to improve both parental mental well-being and neonatal outcomes [41]. In the neonatal unit of this study, KMC was consistently provided throughout hospitalization, which may have mitigated the negative effects of the COVID-19 pandemic, potentially explaining the sustained levels of anxiety and depression [41].

Parental participation did not decrease during T1, as would be expected. We assume that parents who faced difficulties accessing their children in the NICU were less likely to be interviewed, whereas those with access to the NICU were more easily enrolled. In addition, the participation of parents in basic care activities such as diaper changes, feeding, and providing comfort to the baby is a routine practice in our NICU and persisted throughout the pandemic. These factors may explain why no significant difference was found between the two periods.

Interestingly, we identified low correlations between stress (SOL) and both depression and anxiety-trait, suggesting these constructs could represent distinct dimensions of parental mental health in the NICU context. These findings underscore the need for multidimensional mental health assessment in NICU parents, as relying on one single assessment measure could lead to missing important information. Still, we found a strong positive correlation between anxiety trait (STAI-trait) and both depression (EPDS) and anxiety symptoms (EPDS-A), consistent with previous studies demonstrating the depression-related components inherent in the STAI scale [42,43].

Our data collection was carried out during the COVID-19 pandemic, and no other Brazilian data on parental participation is available for comparison, yet overall high levels of participation were observed. IPP scores were higher than those reported in an Italian study with 152 parents [20]. This same study found no significant change in parental stress, depression, or participation in care when comparing the pre-pandemic period with COVID-19 periods [20], suggesting that these levels could remain stable even during peaks of the COVID-19 pandemic and that parental participation in care can be maintained [20].

Previous research has demonstrated that parental participation in care is associated with improved parental mental health outcomes [44]. However, our data analysis failed to identify the correlation between parental participation and parental anxiety, depression, or stress. These findings should be interpreted with caution, as this may reflect limitations inherent to our cross-sectional design, including the inability to assess causality or control for confounders. Future intervention studies should better assess the relationship between parental involvement in neonatal care and parental mental health outcomes.

An important strength of our study is the use of validated scales to measure parental well-being in a quantitative evaluation, which can allow comparative studies in different periods and different settings. Also, it brings a new tool to assess parental participation in neonatal care in future studies.

It is important to underline that this study has some limitations. First, non-standardization of the timing of data collection; depending on the baby’s length of stay in the NICU, different experiences may have occurred, and this could influence the results. Second, low paternal participation may have caused the loss of important information and influenced the results, as some studies show that NICU mothers suffer a greater influence on mental health [45]. The presence of fathers was greatly reduced in the NICU because of the restriction impositions of the COVID-19 pandemic, and future studies at different epidemiological moments should help to fill this gap.

Third, it was not possible to compare our data with the pre-pandemic period, as data collection occurred exclusively during the COVID-19 pandemic period. However, study findings of high levels of mental distress, regardless of the pandemic period, are aligned with existing literature. Since parental psychological distress cannot be fully attributed to the COVID-19 pandemic, assessing stress, anxiety, and depression after the pandemic can help implement strategies to reduce the frequency of these conditions in the NICU. Measures to mitigate the deleterious effects of NICU admission on parents’ mental health should be a priority for healthcare providers. FCC, for instance, is a valuable approach that can be feasible even in low- or middle-income settings. KMC, for example, is widely disseminated in Brazilian neonatal units and offers tools for best practices, including encouraging the presence of parents throughout hospitalization and support for family members [46].

Even though this study focuses on immediate parental mental health challenges, it is also important to assess the long-term consequences of the COVID-19 pandemic burden. Future studies should try to comprehend the long-term psychological effects of the pandemic on parents and the possible influence on their parenting skills.

## 5. Conclusions

Our results add to existing evidence by documenting findings from the Brazilian setting on the level of stress, anxiety, depression, and participation to care among NICU parents. While stress levels were significantly higher during the high COVID-19 incidence period, they remained elevated even in the low COVID-19 incidence period. In contrast, anxiety and depression levels did not significantly change over time. Even though more research is needed to document data beyond the COVID-19 pandemic, these findings underscore the importance of regular monitoring of these parameters. NICU staff need to be sensitive and empowered to manage and prevent psychological distress and develop strategies, such as FCC intervention, to improve parental experience in the NICU. Additionally, this study introduces validated tools to assess parents’ mental health, being a starting point for other Brazilian NICUs to proceed with parental mental health assessment. Prioritizing parental well-being and acknowledging its relevance are key aspects of creating a better NICU environment for both parents and their infants.

## Figures and Tables

**Table 1:** NICU parent characteristics during the COVID-19 pandemic.

Characteristics of Parents	Overall *n* (%)	T1 *n* (%)	T2 *n* (%)	*p*-Value
	N = 106	N = 71	N = 35	
Caregiver				>0.999
Mother	98 (92.5)	66 (93.0)	32 (91.4)	
Father	8 (7.5)	5 (7.0)	3 (8.6)	
Caregiver’s age (years)				**0.046**
18–30	62 (58.4)	43 (60.5)	19 (54.3)	
31–40	34 (32.1)	18 (25.4)	16 (45.7)	
>40	4 (3.8)	4 (5.6)	0 (0.0)	
Marital status				0.872
Married/stable union	80 (75.5)	52 (73.2)	28 (80.0)	
Single/separated	26 (24.5)	19 (26.8)	7 (20.0)	
Place of residence				0.093
City of Recife	27 (25.5)	23 (32.4)	4 (11.4)	
Recife’s metropolitan area	31 (29.2)	20 (28.2)	11 (31.5)	
Rural area/interior	48 (45.3)	28 (39.4)	20 (57.1)	
Years of education				**0.007**
<8 years	23 (21.7)	21 (29.6)	2 (5.7)	
≥8 years	83 (78.3)	50 (70.4)	33 (94.3)	
Occupation				0.769
Unemployed or housewife	65 (61.3)	45 (63.4)	20 (57.2)	
Informal worker	23 (21.7)	14 (19.7)	9 (25.7)	
Formal worker	18 (17)	12 (16.9)	6 (17.1)	
Family income ^1^				0.949
<1 minimum wage	58 (54.7)	39 (55.0)	19 (54.3)	
>1 minimum wage	46 (43.4)	30 (42.2)	16 (45.7)	
Missing	2 (1.9)	2 (2.8)	0 (0.0)	
People that offer support at home				0.572
<3	76 (71.7)	47 (66.2)	29 (82.8)	
≥3	27 (25.5)	21 (29.5)	6 (17.1)	
Missing	3 (2.8)	3 (4.3)	0 (0.0)	

Notes: T1 = high COVID-19; T2 = low COVID-19; ^1^ = minimum wage average in 2020/2021 = USD $215 (19). Bold values indicate statistically significant results (*p* < 0.05).

**Table 2:** Clinical characteristics of newborns during COVID-19 pandemic.

Characteristics of Newborns	Overall *n* (%)	T1 *n* (%)	T2 *n* (%)	*p*-Value
	N = 111	N = 72	N = 39	
Birth Weight in Grams (mean ± SD)	1985 ± 830	2005 ± 880	1822 ± 717	0.241
5′ Apgar				0.653
≥5	5 (4.5)	4 (5.6)	1 (2.6)	
>5	104 (93.7)	66 (91.7)	38 (97.4)	
Missing	2 (1.8)	2 (2.8)	0 (0.0)	
Birth Gestational Age (weeks)				**0.001**
≤27	6 (5.4)	6 (8.3)	0 (0.0)	
28–33	57 (51.4)	36 (50.0)	21 (53.8)	
34–36	19 (17.1)	6 (8.3)	13 (33.3)	
37–41	29 (26.1)	24 (33.3)	5 (12.8)	
Resuscitation at Birth ^1^				>0.999
Yes	44 (39.6)	29 (40.3)	15 (38.5)	
No	67 (60.4)	43 (59.7)	24 (61.5)	
Congenital Malformation ^2^				0.384
Yes	32 (28.8)	23 (31.9)	9 (23.1)	
No	79 (71.2)	49 (68.1)	30 (76.9)	
Unit of Admission				0.490
NICU	102 (91.9)	65 (90.3)	37 (94.9)	
Semi-intensive care	9 (8.1)	7 (9.7)	2 (5.1)	
Intubation				0.664
Yes	33 (29.7)	20 (27.8)	13 (33.3)	
No	78 (70.3)	52 (72.2)	26 (66.7)	
Parenteral Nutrition				0.664
Yes	33 (29.7)	20 (27.8)	13 (33.3)	
No	78 (70.3)	52 (72.2)	26 (66.7)	
Surgery ^3^				>0.999
Yes	20 (18.0)	13 (18.1)	7 (17.9)	
No	91 (82.0)	59 (81.9)	32 (82.1)	
Suspected COVID-19 ^4^				0.111
Yes	58 (52.2)	42 (58.3)	16 (41.0)	
No	53 (47.7)	30 (41.7)	23 (59.0)	
Confirmed COVID-19 ^5^				>0.999
Yes	10 (9.0)	7 (9.7)	3 (7.7)	
No	101 (91.0)	65 (90.3)	36 (92.3)	
Total Hospital Stay (mean ± SD)	32.6 ± 23.6	32.5 ± 24.9	32.6 ± 21.3	0.983

Notes: The table shows absolute frequencies and percentages unless otherwise specified. T1 = high COVID-19; T2 = low COVID-19; ^1^ = use of positive pressure ventilation at birth, ^2^ = malformation associated with clinical impairment, ^3^ = surgery (as described in medical record), ^4^ = negative COVID-19 swab, ^5^ = positive result of COVID-19 swab. Abbreviations: NICU = neonatal intensive care; SD = standard deviation. Bold values indicate statistically significant results (*p* < 0.05).

**Table 3:** Stress, anxiety, depression, and parental participation in NICU parents during the COVID-19 pandemic.

Assessment Instrument	Overall	T1	T2	*p*-Value
**PSS:NICU**	N = 106	N = 71	N = 35	
**Overall Stress**—OSL ≥ 3, *n (%)*	28 (26.4)	17 (23.9)	11 (31.14)	0.484
Sights and sounds, *median [IQR]*	1.83 [1.17, 2.5]	1.67 [1.17, 2.42]	1.83 [1.17, 2.5]	0.888
Infant behavior and appearance, *median [IQR]*	1.85 [1.23, 2.85]	1.85 [1.31, 2.77]	1.54 [1.15, 3.23]	0.768
Parental role alteration, *median [IQR]*	3.29 [1.86, 4.54]	3.57 [2.07, 4.57]	3.14 [1.0, 4.14]	0.071
OSL total score, *median [IQR]*	2.27 [1.62, 3.03]	2.27 [1.65, 2.94]	2.27 [1.29, 3.13]	0.588
**Stress Occurrence**—SOL ≥ 3, *n (%)*	55 (51.9)	40 (56.3)	15 (42.9)	0.298
Sights and sounds, *median [IQR]*	2.17 [1.50, 3.37]	2.2 [1.50, 3.67]	2.08 [1.50, 2.92]	0.222
Infant behavior and appearance, *median [IQR]*	2.82 [2.00, 3.91]	2.88 [2, 4.28]	2.82 [2.0, 3.67]	0.249
Parental role alteration, *median [IQR]*	4.00 [2.58, 4.86]	4.14 [3.00, 5.00]	3.29 [1.93, 4.67]	**0.046**
Total Score, *median [IQR]*	3.05 [2.2, 3.92]	3.24 [2.28, 4.04]	2.68 [1.84, 3.43]	**0.021**
**STAI Trait**	N = 104	N = 69	N = 35	
Anxiety—STAI trait > 40, *n (%)*	69 (66.3)	45 (63.4)	24 (68.6)	0.828
Anxiety—STAI trait, *mean* (SD)	44.0 (8.8)	44.0 (8.8)	44.1(8.7)	0.969
**EPDS-A**	N = 106	N = 71	N = 35	
Anxiety EPDS-A ≥ 6, *n (%)*	30 (28.3)	21 (29.6)	9 (35.7)	0.819
**EPDS**	N = 106	N = 71	N = 35	
Depression—EPDS ≥ 13, *n (%)*	35 (33.0)	24 (33.8)	11 (31.4)	0.999
EPDS total score, *median [IQR]*	10 [6.0, 15]	10 [6.0, 15]	10 [6.5, 14]	0.671
**IPP**	N = 106	N = 71	N = 35	
Activities related to daily living (range: 0–6), *median [IQR]*	4.0 [2.0, 5.0]	4.0 [2.0, 5.0]	5 [3.0, 6.0]	0.139
Providing comfort (range: 0–7), *median [IQR]*	6.0 [5.0, 7.0]	6.0 [5.0, 7.0]	6.0 [5.0, 7.0]	0.772
Advocation (range: 0–7), *median [IQR]*	5.0 [4.0, 6.0]	5.0 [4.0, 6.0]	5.0 [4.0, 6.0]	0.390
Technical task (range: 0–10), *median [IQR]*	6.0 [4.0, 8.0]	6.0 [3.5, 7.0]	7.0 [4.0, 9.0]	0.300
IPP total score (range: 0–30), *median [IQR]*	19.39 [15.0, 24.7]	20 [13.5, 24]	23 [18.0, 25.0]	0.235

Notes: T1 = high COVID-19; T2 = low COVID-19. Abbreviations: IQR = inter-quartile range; PSS:NICU = Parental Stressor Scale in NICU; OSL = Overall Stress Level; SOL = stress occurrence level; STAI = State-Trait Anxiety Inventory; EPDS-A = Edinburgh Postnatal Depression Scale Anxiety; EPDS = Edinburgh Postnatal Depression Scale; IPP = Index of Parental Participation/Hospitalized Infant. Bold values indicate statistically significant results (*p* < 0.05).

## Data Availability

Data transmission and storage were secured by encryption. Data supporting the conclusions of this article is available in REDCap and can be shared by the authors on request.

## Supplementary Materials

The following supporting information can be downloaded at https://www.mdpi.com/article/10.3390/children12040496/s1: Figure S1: Translation and cultural adaptation of the Index of Parental Participation/Hospitalized Infant (IPP-HI) [47]; Figure S2: Flow diagram of parental eligibility and consent; Table S1: Strengthening the Reporting of Observational Studies in Epidemiology guideline (STROBE); Table S2: Key characteristics of questionnaire used for data collection.
